# The effect of gender and age on the association between weight status and health-related quality of life in Australian adolescents

**DOI:** 10.1186/1471-2458-14-898

**Published:** 2014-09-01

**Authors:** Kristy Bolton, Peter Kremer, Naomi Rossthorn, Marj Moodie, Lisa Gibbs, Elizabeth Waters, Boyd Swinburn, Andrea de Silva

**Affiliations:** WHO Collaborating Centre for Obesity Prevention, Deakin University, Geelong, Australia; School of Exercise and Nutrition Sciences, Deakin University, Geelong, Australia; School of Psychology, Deakin University, Geelong, Australia; Deakin Health Economics, Deakin University, Burwood, Australia; Jack Brockhoff Child Health and Wellbeing Program, Melbourne School of Population and Global Health, The University of Melbourne, Carlton, Australia; School of Population Health, University of Auckland, Auckland, New Zealand; Dental Health Services Victoria, Carlton, Australia; Melbourne Dental School, The University of Melbourne, Carlton, Australia

**Keywords:** Health-related quality of life, Weight status, Age, Gender, Adolescents, Obesity

## Abstract

**Background:**

Evidence suggests an inverse relationship between excess weight and health-related quality of life (HRQoL) in children and adolescents, however little is known about whether this association is moderated by variables such as gender and age. This study aimed to investigate these relationships.

**Methods:**

Participants were secondary school students (818 females, 52% and 765 males, 48%) from 23 secondary schools in Victoria, Australia. Age ranged from 11.0 to 19.6 years (mean age 14.5 years). The adolescent version of the Assessment of Quality of Life (AQoL) Instrument (AQoL-6D) which is a self-reported measure of adolescent quality of life was administered and anthropometric measures (height and weight) were taken. Assessment of weight status was categorized using the Body Mass Index (BMI).

**Results:**

HRQoL was associated with gender and age, but not weight status or socio-economic status; with males and younger adolescents having higher HRQoL scores than their female and older adolescent counterparts (both p < 0.05). There was also a significant interaction of weight status by gender whereby overweight females had poorer HRQoL (-.06 units) relative to healthy weight females (p < 0.05).

**Conclusions:**

This study contributes to the evidence base around factors associated with adolescent HRQoL and reveals that gender and age are important correlates of HRQoL in an Australian adolescent population. This knowledge is critical to inform the design of health promotion initiatives so they can be tailored to be gender- and age-specific.

**Trial registration:**

Australian Clinical Trials Registration Number 12609000892213.

## Background

Obesity is a major health concern. Globally, it has been estimated that 10% of children and adolescents aged five to 17 years old are overweight and, of these, two to three per cent are obese [1]. The most recent data in Australia (2011-2012) revealed the prevalence of overweight and obesity in Australian adults has increased to 63.4% (35.0% overweight, 28.3% obese), and children aged 5-17 years to 25.3% (17.7% overweight, 7.6% obese) [2]. The health implications of obesity include the development of heart disease, cardiovascular disease, hypertension, type 2 diabetes and musculoskeletal problems due to the mechanical stress on the body [3–5].

Obesity contributes to the global burden of chronic disease and disability and has been found to be associated with social, economic and cultural factors and satisfaction with life [5, 6]. Consequences of obesity also extend to psychological and social aspects of well-being [7] which also are vital to good health. The World Health Organisation Constitution states that health is not merely the absence of disease or infirmity, but a state of complete physical, mental and social well-being [8]. Functional status and well-being is commonly referred to as health-related quality of life (HRQoL) [9] and the impact of diseases (such as obesity), environmental and economic factors such as income and education can all influence HRQoL [10].

HRQoL is a multidimensional measure based upon an individual’s satisfaction or happiness in various life domains that affect or are affected by health [11]. Factors such as weight status, age, gender can affect HRQoL [7, 9, 12]. With regards to an individual’s weight status, recent research in adult populations has suggested that obesity impacts negatively on functional health and well-being (HRQoL) [9, 10]. Research has expanded to child and adolescent populations and supports the associations found in adult populations whereby poorer HRQoL was experienced by children and adolescents with excess weight [6, 13–18]. Furthermore, studies examining gender effects on HRQoL have revealed female children and adolescents to report lower HRQoL in comparison to their male counterparts [7, 11, 19–21]. Studies have also revealed an association between increasing age and poorer HRQoL scores across 12 European countries [11], greater physical and psychological well-being in children compared to adolescents [19] and evidence to suggest the higher the age, the lower the HRQoL scores in adolescents [7].

In recent studies, many variables affecting HRQoL are beginning to be examined together. Gender influences the association of HRQoL and weight status, with females with excess weight having lower HRQoL [7, 12, 21]. A relationship has also been observed between HRQoL and weight status as children and adolescents age with younger overweight adolescents reporting significantly lower HRQoL scores [12]. This pattern has also been observed in students with obesity whereby younger students with obesity have higher HRQoL compared to older students with obesity [16, 22]. Subsequent studies analysing the effect of age further, suggest the association of lower HRQoL and obesity is weak and/or absent in very young children (aged 2-5 years) but appears more in school years, and steadily strengthens with age [18].

Evidence suggests an association between HRQoL and weight status, however less is known regarding gender and age as moderating factors on the association between weight status and HRQoL. HRQoL and BMI may track strongly longitudinally in children growing into adolescents [23]. This is a concern and suggests we need to understand the issues and subsequently intervene early in the life-stage to avoid the development of overweight and obesity, the potential associated chronic health conditions and poorer HRQoL. Much of the research already conducted examining the effects of weight status has largely documented the impacts on adults and children and to a lesser extent, defined adolescents as a cohort separate to children [6, 15, 17, 24]. There are distinct changes occurring during the growth between a child and adolescent; namely the physical and psychological changes accompanying the onset of puberty [25–27]. Therefore it is essential to separately assess how children and adolescents perceive their own situation [11] and examine any differences in HRQoL as they age.

The present study aims to build the evidence base by investigating 1) the association of weight status (healthy weight vs overweight and obese), gender and age (younger vs older adolescents); on self-reported HRQoL; and 2) examine whether the association of weight status on HRQoL is moderated by either age or gender among a sample of Australian adolescents.

## Methods

### Participants

Participants consisted of 1583 secondary school students recruited from 23 schools in various communities across Victoria, Australia (818 female (51.7%) and 765 male (48.3%)) [28]. The participants were aged from 11.0 to 19.6 years (mean age14.5, SD = 1.5 years). Schools in the current study were part of a larger health promoting study and selected for involvement as previously described [28]. Briefly, schools within intervention communities were invited to participate in the study, and subsequently comparison schools selected using stratified random sampling to match intervention school demographics such as school type, school size, level of disadvantage and location [28]. This study utilises baseline data only, consequently intervention or comparison status of schools is irrelevant. Parents provided written consent and participants provided verbal consent prior to data collection. Approval for this study was granted by the Deakin University Human Research Ethics Committee (EC98-2008), the Department of Education and Early Childhood Development and relevant Catholic dioceses where appropriate. The project was registered with the Australian Clinical Trials (registration number 12609000892213).

### Materials and apparatus

#### Demographics

A combined plain language statement and consent form were used to obtain information about age, gender, Aboriginal and/or Torres Strait Islander ethnicity, migration status and residential postcode which was used to calculate socio-economic status (SES). The 2006 Census data was used to determine the Socio-Economic Index For Areas (SEIFA) score on the index of relative socio-economic disadvantage [29, 30]. This area-level index is based on data collected from the 2006 Australian census of population and housing, and incorporates variables such as income, education, occupation, living conditions, access to services and wealth. A lower score on the index indicates that an area is more disadvantaged [31].

#### Health-related quality of life

Participants were asked to complete the adolescent version of the Assessment of Quality of Life (AQoL) AQoL-6D which measures adolescent HRQoL. Developed in Australia, the AQoL-6D adolescent survey is an adapted version of the AQoL 2 designed for and validated in adults [32, 33]; the utility weights have been recalibrated for adolescents [34]. The AQoL theoretical framework was based on the effects of ill health on a person’s capacity to function; the health descriptions were established using the WHO’s disabilities and impairments framework [32, 35]. This self-reported instrument consists of 20 items that produce scores on six domains. Each domain is measured by three to four items pertaining to that domain; physical ability (4 items), social and family relationships (3 items), mental health (4 items), coping (3 items), pain (3 items) and vision, hearing and communication (3 items) [36].

#### Anthropometry

Height and weight were measured and recorded as previously defined [28]. Briefly, weight and height was measured by trained research staff in a private and sensitive manner behind screens. Each measurement was taken twice, and a third measurement was only taken if the first two measurements were outside defined parameters as previously reported [28]. Heavy clothing and shoes were removed prior to measurement. Weight was recorded to the nearest 0.1 kilogram using calibrated digital scales. Height was recorded to the nearest 0.1 centimeter, using a portable stadiometer with a movable headboard that lowered to touch the crown of the head [28]. BMI was calculated using weight in kilograms divided by height in metres^2^ (kg/m^2^). Standardized BMI scores were used to categorize weight status into healthy, overweight/obese categories using the World Health Organisation Growth Reference for 5- to 19-year-old children BMI cutoff values [37]. The thin category was excluded from the dataset due to low numbers (n = 4).

### Data treatment and analysis

Data were double entered by research staff. Data were cleaned and analysed using Stata 10.0.

#### AQoL-6D

Weighted item scores from the 20 questions were combined to form dimension scores that were added into a single multiplicative score using a scoring algorithm [38]. This algorithm includes a specific adjustment of the overall single multiplicative score for participants who are Australian adolescents [34].

#### Coding of variables

The age variable was dichotomized into younger adolescents (11.00 to 14.99 years) and older adolescents (≥15.00 to 19.00 years) [25].

Descriptive data were summarised as means with standard deviations (±SD), or proportions for total population and for male and female subgroups to describe characteristics of the sample. Associations between key demographic variables were tested using Chi-square tests. Separate univariate ANOVAs were used to test for significant differences in AQoL by weight status, gender and age group. Multiple linear regression (MLR) analysis was also used to test for associations between weight status and AQoL score and effects are reported as unstandardized coefficients (B). Three MLR models were tested: model 1 tested for associations with weight status; model 2 also tested for associations with weight status but with gender, age and area-SES covariates included; model 3 included same the covariates as model 2 but also included the interaction terms of weight status by gender and weight status by age. All models were adjusted for clustering by school. P < 0.05 was considered statistically significant. Note that demographics and surveys were collected from 1583 students however anthropometric measurements were taken from 944 students as indicated by the sample numbers displayed in tables. Two rounds of data collection occurred at each school. Round 1 involved collecting demographic information, survey (AQoL6-D) and anthropometric data from participating students. Due to school-related limits on student access for data collection in round 2, it was only possible to collect demographic information and survey data from these participating students. As data was collected from the same schools at both time points, the characteristics of the sample at round 1 and round 2 are similar.

## Results

Characteristics of the adolescent sample are shown in Table 1. Over two-thirds of the student population were <15 years old and approximately one quarter were overweight or obese. The majority of students were born in Australia and only a small proportion were of Aboriginal and/or Torres Strait Islander origin. Over three-quarters of the students were from areas classified into the two lower SES quartiles (<50^th^ percentile).

**Table 1:** **Demographic profile of adolescent sample (n (proportion%))**

	Total	Male	Female
	n = 1583	n = 765 (48.3%)	n = 818 (51.7%)
**Age**			
Younger (<15 years)	999 (63.1%)*	450 (58.8%)	549 (67.1%)
Older (≥15 years)	548 (36.9%)	315 (41.2%)	269 (32.9%)
**Weight status**			
Healthy weight	727 (75.0%)	340 (73.1%)	387 (76.6%)
Overweight/obese	243 (25.0%)	125 (26.9%)	118 (17.4%)
**Newly arrived**			
Not newly arrived	1508 (95.2%)	729 (95.3%)	779 (95.2%)
Arrived 5-10 years ago	31 (2.0%)	14 (1.8%)	17 (2.1%)
Arrived < 5 years ago	44 (2.8%)	22 (2.9%)	22 (2.7%)
**Indigenous and/or Torres Strait Islander origin**			
Yes	27 (1.9%)	10 (1.5%)	17 (2.4%)
No	1384 (98.1%)	680 (98.5%)	704 (97.6%)
**Socio-economic status** ^**^**^			
1 high disadvantage	638 (40.3%)^§^	271 (35.4%)	367 (44.9%)
2	572 (36.1%)	281 (36.7%)	291 (35.6%)
3	191 (12.1%)	106 (13.9%)	85 (10.4%)
4 low disadvantage	182 (11.5%)	107 (14.0%)	75 (9.1%)

^^^SES has been categorized into quartiles, based on SEIFA.

*p = 0.001 by Chi2 test in total sample; ^§^p < 0.001 by Chi2 test in total sample.

The mean total HRQoL for healthy weight, overweight/obese adolescents (overall and stratified by gender and age category) are displayed in Table 2. Results of ANOVA revealed a significant difference in HRQoL for gender and age; males and younger adolescents had higher self-reported HRQoL than their female and older adolescent counterparts.

**Table 2:** **Mean (SD) AQoL score for weight status category, gender and age**

Variable	n	Mean AQoL (SD)	F	p
**All**	1404	0.88 (0.14)	*-*	*-*
**Weight status**				
Healthy weight	710	0.89 (0.14)	3.26	0.07
Overweight/obese	234	0.87 (0.14)		
**Gender**				
Male	682	0.89 (0.13)	9.68	0.002
Female	720	0.87 (0.15)		
**Age**				
Younger (<15 years)	889	0.89 (0.14)	13.73	<0.001
Older (≥15 years)	515	0.86 (0.14)		

SD: standard deviation.

Note: sample size varies due to demographic and survey data being collected from all participating students, and anthropometric data from n = 944 students only.

Results of the multiple linear regression analyses are shown in Table 3. There was no association between weight status and self-reported HRQoL (model 1), even when the gender, age and area-SES covariates were included (model 2); gender and age were associated with HRQoL however area-SES or weight status was not. Similarly, in model 3, which included the two interaction terms, weight status was not associated with HRQoL. Of the covariates, gender and area-SES were not associated but age was associated with HRQoL. The interaction of weight status by age was not associated with HRQoL but the interaction of weight status by gender was; overweight females had significantly poorer HRQoL (-.06 units) relative to healthy weight females.

**Table 3:** **Multiple linear regression models for associations between weight status and HRQoL, with gender and age**

	Model 1		Model 2		Model 3	
	n = 944		n = 889		n = 889	
**Variables**	**B coef (SE)**	**95% CI**	**B coef (SE)**	**95% CI**	**B coef (SE)**	**95% CI**
**Weight status** (ref: healthy weight)	-0.02 (0.01)	-0.05, 0.01	-0.02 (0.01)	-0.05, 0.01	0.00 (0.01)	-0.02, 0.04
**Gender** (ref: male)			-0.03 (0.01)	-0.05, 0.00*	-0.01 (0.01)	-0.04, 0.01
**Age** (ref: younger)			-0.02 (0.01)	-0.04, 0.00^#^	-0.02 (0.01)	-0.04, 0.00^^^
**Socio-economic status** (ref: low disadvantage)			0.00 (0.00)	0.00, 0.00	0.00 (0.00)	0.00, 0.00
**Weight status by gender** (ref: healthy weight males)					-0.06 (0.03)	-0.11, -0.00^§^
**Weight status by age** (ref: healthy weight younger)					-0.00 (0.03)	-0.06, 0.06

Regression B coefficients (unstandardized) represent differences in total AQoL score compared with reference group (ref). All models adjusted for clustering by school (n = 23). Model 2 and 3 were also adjusted for socio-economic status. *p = 0.026, ^#^p = 0.022, ^^^p = 0.046, ^§^p = 0.045.

## Discussion

The purpose of this study was to investigate the association of weight status, gender and age on self-reported HRQoL, and to examine whether the association of weight status on HRQoL is moderated by either gender or age in a sample of Australia adolescents. The findings from this study indicate that individual variables such as gender and age affect self-reported HRQoL in an Australian adolescent population. In contrast, there was no relationship between weight status and HRQoL. However when gender was added to the model examining weight status and HRQoL, a significant interaction was discerned whereby gender moderated the association between weight status and HRQoL. Specifically, the interaction indicated that relative to males, females who were overweight had significantly poorer HRQoL compared to healthy weight females.

### Weight status and HRQoL

The lack of associations regarding weight status in this study could be due to the HRQoL assessment tool utilized - perhaps AQoL-6D is less sensitive to weight-related variations in HRQoL, and comparison of common HRQoL tools and AQoL-6D in the same population would be interesting to further investigate. Relationships between weight status and particular domains of HRQoL assessment tools have been demonstrated [6, 18]. Not only do the six AQoL-6D domains differ to other commonly utilised HRQoL tools such as PedsQL, SF36 and KIDSCREEN52, but analysis of the AQoL-6D domains is not currently possible. This tool has currently only been weighted in an adolescent population for the overall score, not the individual domains (which are adult weighted) [34]. However if domain analysis was possible, associations between weight status and domains could possibly be identified.

The lack of an association between weight status and HRQoL is in contrast to other studies that have suggested that overweight and obese adolescents report lower HRQoL compared to those healthy weight children and adolescents [9, 12, 13, 15–18, 39–41] and that increasing weight status negatively impacts overall paediatric HRQoL [9]. There are three studies which have not reported a significant association between weight status and HRQoL [42–44] however potential reasons why this was the case were not discussed by the authors. The systematic review by Tsiros et. al [9] which examined weight status and HRQoL also had the limitation of including clinical treatment seeking populations which may have resulted in an overestimation of the strength of the associations presented [23] and lack generalisability to the population [12]. The fact that previous studies have reported an association while we did not might reflect a number of methodological differences; specifically the different HRQoL assessment tools utilised (e.g. self-report and parent-report); different sample sizes and characteristics (e.g. small sample sizes, clinical treatment seeking populations); and the methodology surrounding anthropometric measurements (e.g. self-reported, parent-reported measurements).

Environmental, economic and cultural factors can substantially affect well-being [10, 11] and could also be an explanation to the lack of significant association between weight status and HRQoL in the current study. For example, a meaningful negative association between excess weight and HRQoL was unable to be demonstrated in a population of Fijian students possibly due to socio-economic and socio-cultural factors [22]. The current sample population in this study was socio-economically disadvantaged, included some large rural and regional areas, and some communities we culturally and linguistically diverse – all potential influencing factors which might explain a lack of an association and require further investigation. Additionally, it could be speculated that perhaps these communities have different social norms, or perhaps community efforts to support and not stigmatise overweight and obesity have been effective hence no meaningful association between excess weight and HRQoL. However, despite the cultural differences between Fiji and Australia, it is intriguing that the findings regarding a lack of association between weight status and HRQoL in Fiji are supportive and strengthen the current study findings in this adolescent population in Australia. Given that the association between excess weight and HRQoL became significant once gender was added into the model, perhaps there is some other unknown factor complicating this relationship yet to be determined.

### Gender and HRQoL

This study revealed significant differences in HRQoL for male and female adolescents, with females reporting lower HRQoL. Various reasons have been proposed to explain this association in the literature and include the notion that puberty is physically more extreme for females (e.g. menstruation), females have varied coping mechanisms (coping patterns are inwards for females, outwards for males), puberty hormones, social demands which can be difficult to achieve, the influence of traditional female stereotypes, confronting beauty ideals [19], and greater body image concerns [21]. Bonsergent et.al (2012) proposes that girls may be more attuned and aware of their bodies compared to males; and seek to be thin and fit due to the ideal body shape demonstrated in television, advertising, magazines and social stigmas attached to obesity [7]. Many studies support the finding of lower HRQoL for females [7, 11, 19–21].

### Age and HRQoL

Older adolescents in this study reported lower levels of HRQoL than younger adolescents. Differences in HRQoL as children and adolescents age could be resultant from the physical and social transition experienced as they grow and age; particularly if combined with transitioning to new schools [19]. Adolescents undergo a process of individuation and autonomy that is very important to them [19]. Adolescents begin to develop their own values and cultural norms; and are being challenged with new developmental tasks, and a vast number of new experiences including being socially accepted as peers become more important than adults [19]. Bonsergent et. al (2012) found decreasing HRQoL with increasing age [7] which was further supported by a European multi-country study which demonstrated better HRQoL values in children compared to adolescents [11].

Whilst there was no significant overall association for weight status on HRQoL (model 2); when the weight status-HRQoL association was modelled in conjunction with gender (model 3), a more complex association was found which suggests that gender is an important moderating factor and strengthens the relationship between weight status and HRQoL. The association for gender found in model 2 was not significant when the combination term was included (model 3). Together this complex set of findings suggests that the association of weight status on HRQoL is particularly salient for adolescent females but of little impact to males. Other studies that have reported similar findings regarding gender [7, 12, 21]. Keating (2011) reported that whilst both sexes experience significant decreases in HRQoL associated with obesity, the effect was doubled in magnitude for females [39]. This association can possibly be explained by the different hormones female experience during puberty, their inward coping mechanisms, social demands and influences of traditional female stereotypes, beauty ideals [19] and body image concerns [21].

With regards to the association between weight status and HRQoL in conjunction with age we found that age did not moderate this relationship. This could possibly be due to differing tools to assess HRQoL; and differing sample populations (i.e. treatment seeking populations which limits generalisability to the population). However, previous research has indicated that age play an important role in moderating the association between weight status and HRQoL [9, 12, 18]. It has been suggested that early adolescence (<14 years) is a particularly vulnerable period for decreases in HRQoL in overweight/obese adolescents potentially due to emotional development and awareness of social exclusion [9].

### Limitations and future directions

This study adds value to population data and trends for adolescent health in Australia where little is known regarding the associations of different factors on adolescent self-reported HRQoL. A particular strength of the study is the large sample size of adolescent females and males which were not treatment seeking individuals, but sampled from the general population. It employed a widely used, psychometrically tested and validated Australian based psychometric instrument to measure self-reported HRQoL which has been specifically calibrated and validated in adolescents [34, 39]. This research focus on an adolescent population further adds to the development of Australian population norms and health research into effects of obesity.

Whilst this study extends current Australian health research into obesity and HRQoL focusing specifically on the developmental phase of adolescence, we acknowledge several study limitations. Whilst the methodology limits the generalisability to other Australian studies that have investigated children [17, 24] given the different tools utilised to collect HRQoL, the study findings still highlight the importance of investigating these moderating factors further. The self-reported and cross-sectional nature of the data is also a limitation. Future research could include exploring potential associations between HRQoL and subgroups of excess weight (i.e. analysing overweight and obese categories separately) and in-depth examination of family and socio-economic patterns to determine any influences on HRQoL and weight status such as household finances, familial eating patterns, lifestyle behaviors and relationships. Longitudinal studies would be particularly beneficial to track HRQoL changes over time. Several of the authors on this study have previously demonstrated the effect of ethnicity on child overweight and obesity over and above socio-economic status [45]. Exploration of ethnic diversity and body image to better understand HRQoL in a cultural context would continue to add to diversity and population norms.

## Conclusion

The present investigation found that females had lower HRQoL compared to males, and lower HRQoL was reported for older compared to younger adolescents. Additionally, in this adolescent population, gender acted as a significant moderator on weight status and HRQoL, subsequently overweight females had poorer HRQoL compared to healthy weight females. More understanding of these associations from longitudinal studies would shed light on the temporal nature of these types of associations, their causal pathways and specific mechanisms. [18] Information from this study will help inform the design of health promotion initiatives so they can be tailored to be gender- and age-specific. Further research into adolescent HRQoL and weight status is beneficial in developing targeted health promotion programs that incorporate evidence-based interventions for adolescents who are in the critical stage of establishing poor lifestyle behaviours and are at risk of developing obesity. Promoting a normal body weight has the potential to improve health and well-being in the young, and affect the risk of disease later in life [18].

## Abbreviations

AQoL: Assessment of quality of life
HRQoL: Health-related quality of life
QoL: Quality of life
SEIFA: Socio-economic indexes for areas
SES: Socio-economic status
PedsQL: Pediatric Quality of Life Inventory.
